# Phage Biocontrol of *Pseudomonas aeruginosa* in Water

**DOI:** 10.3390/v13050928

**Published:** 2021-05-17

**Authors:** Ari Kauppinen, Sallamaari Siponen, Tarja Pitkänen, Karin Holmfeldt, Anna Pursiainen, Eila Torvinen, Ilkka T. Miettinen

**Affiliations:** 1 Expert Microbiology Unit, Department of Health Security, Finnish Institute for Health and Welfare, FI-70701 Kuopio, Finland; sallamaari.siponen@uef.fi (S.S.); tarja.pitkanen@thl.fi (T.P.); anna.pursiainen@thl.fi (A.P.); ilkka.miettinen@thl.fi (I.T.M.); 2 Department of Environmental and Biological Sciences, University of Eastern Finland, FI-70211 Kuopio, Finland; eila.torvinen@uef.fi; 3 Department of Food Hygiene and Environmental Health, Faculty of Veterinary Medicine, University of Helsinki, FI-00014 Helsinki, Finland; 4 Centre for Ecology and Evolution in Microbial Model Systems (EEMiS), Department of Biology and Environmental Sciences, Linnaeus University, SE-39231 Kalmar, Sweden; karin.holmfeldt@lnu.se

**Keywords:** *Pseudomonas aeruginosa*, bacteriophages, biocontrol, phage cocktail, water treatment

## Abstract

Bacteriophage control of harmful or pathogenic bacteria has aroused growing interest, largely due to the rise of antibiotic resistance. The objective of this study was to test phages as potential agents for the biocontrol of an opportunistic pathogen *Pseudomonas aeruginosa* in water. Two *P. aeruginosa* bacteriophages (vB_PaeM_V523 and vB_PaeM_V524) were isolated from wastewater and characterized physically and functionally. Genomic and morphological characterization showed that both were myoviruses within the *Pbunavirus* genus. Both had a similar latent period (50–55 min) and burst size (124–134 PFU/infected cell), whereas there was variation in the host range. In addition to these environmental phages, a commercial *Pseudomonas* phage, JG003 (DSM 19870), was also used in the biocontrol experiments. The biocontrol potential of the three phages in water was tested separately and together as a cocktail against two *P. aeruginosa* strains; PAO1 and the environmental strain 17V1507. With PAO1, all phages initially reduced the numbers of the bacterial host, with phage V523 being the most efficient (>2.4 log_10_ reduction). For the environmental *P. aeruginosa* strain (17V1507), only the phage JG003 caused a reduction (1.2 log_10_) compared to the control. The cocktail of three phages showed a slightly higher decrease in the level of the hosts compared to the use of individual phages. Although no synergistic effect was observed in the host reduction with the use of the phage cocktail, the cocktail-treated hosts did not appear to acquire resistance as rapidly as hosts treated with a single phage. The results of this study provide a significant step in the development of bacteriophage preparations for the control of pathogens and harmful microbes in water environments.

## 1. Introduction

The control of harmful bacteria is an increasingly important and topical public health issue as antibiotic-resistant and disinfection-tolerant pathogenic strains become more common. Bacteriophages, which are viruses that exclusively infect bacteria, play an important role in biological processes by controlling the natural balance in an ecosystem, and hold great potential for targeting harmful bacteria. For biocontrol applications, promising candidates are broad-host-range lytic phages, which destroy the infected cell after immediate replication of the virion. One of the benefits of using bacteriophages over antibiotics or disinfectants is their specificity, i.e., they can target just the pathogenic bacteria of interest, rather than all bacteria. Currently, bacteriophages have mainly been tested in clinical settings [1,2] and environmental applications are still rare. However, the interest in phages has recently increased, e.g., in agriculture [3], aquaculture [3,4] and the food industry [5,6,7]. Biocontrol using bacteriophages may also have potential in several applications in water environments, such as for the mitigation of pathogens, including antibiotic-resistant strains, as well as harmful microbes responsible for causing problematic corrosion and biofouling [8,9,10,11,12,13].

The prevalence of antibiotic resistance (AR) has increased and become more versatile in past years, and currently AR is one of the most significant health threats to modern society [14,15]. The environment may play an important role in the spread of AR and water can act as a vector, enabling the spread of AR properties [16,17]. Currently, antibiotic-resistant bacterial strains have been described for several opportunistic pathogens, including multidrug-resistant *P. aeruginosa*. This Gram-negative bacterium is an opportunistic pathogen that is capable of causing acute and chronic infections of wounds, eyes, ears and respiratory and urinary tracts, particularly among immunocompromised people [18]. Metabolic versatility, the ability to form biofilms and drug/chemical resistance are characteristics enabling the success of *P. aeruginosa* in clinical and environmental settings, including in water [19,20,21].

The role of *P. aeruginosa* in outbreaks related to contaminated tap or bottled water has been well described [22,23,24,25,26]. Moreover, *P. aeruginosa* has been frequently isolated in the water of swimming pools and spas [27,28], and several outbreaks have been described [29,30]. *P. aeruginosa* benefits from the warm and moist conditions present in swimming pools and spas, and it can tolerate chlorination (<1 mg/L) to some extent [30]. Although chlorination is a widely used disinfection method in water environments, the excessive chlorination required to control *P. aeruginosa* has several drawbacks, such as the formation of harmful disinfection by-products. Furthermore, chlorination is relatively ineffective against microbes protected by biofilms, and it may compromise system performance if used, e.g., in biological water treatment processes [12]. Moreover, previous studies have indicated that chlorine exposure may promote the development of AR in bacteria [31,32]. Therefore, new control options are needed and biocontrol using bacteriophages is one of the promising alternatives.

In this study, three *P. aeruginosa* phages were tested against two *P. aeruginosa* strains in order to determine their potential as biocontrol agents in water. The results of this study provide insights into potential future applications in water treatment and reuse.

## 2. Materials and Methods

### 2.1. P. aeruginosa Strains and Growth Conditions

A *P. aeruginosa* PAO1 (DSM 19880) strain, purchased from the Leibniz Institute DSMZ (German Collection of Microorganisms and Cell Cultures GmbH, Braunschweig, Germany), and the environmental strain *P. aeruginosa* 17V1507, isolated from swimming pool water, were used for this study. *P. aeruginosa* 17V1507 was extracted from a swimming pool water sample according to EN ISO 16266:2008 and confirmed using an API 20 NE test (Biomerieux, Marcy-l’Étoile, France). *P. aeruginosa* was propagated in a tryptic soy broth (TSB) and agar (TSA) for 21 ± 3 h, at 36 °C ± 2 °C.

### 2.2. Phages and Growth Conditions

A *Pseudomonas* phage JG003 isolate (DSM 19870), purchased from the Leibniz Institute DSMZ (German Collection of Microorganisms and Cell Cultures GmbH, Braunschweig, Germany), was used for this study. In addition, two environmental strains (*Pseudomonas* phage vB_PaeM_V523 and *Pseudomonas* phage vB_PaeM_V524) isolated from wastewater using *P. aeruginosa* PAO1 (DSM 19880) as a host (see isolation procedures below) were exploited. *P. aeruginosa* phages were enumerated using a double agar layer (DAL) method [33] from serially diluted samples using PAO1 (DSM 19880) as a host. DAL-plates (1.5% TSA bottom agar with 0.7% TSA top agar) were incubated for 21 ± 3 h at 36 °C ± 2 °C.

### 2.3. Phage Isolation and Purification

Two environmental *P. aeruginosa* phage strains (*Pseudomonas* phage vB_PaeM_V523 and *Pseudomonas* phage vB_PaeM_V524) were isolated from the influent of a municipal wastewater treatment plant (Kuopio, Finland). A 100 mL wastewater sample was centrifuged at 9000× *g* for 10 min at 4 °C and the supernatant was filtered through 0.45-µm (Acrodisc, Pall Corporation, New York, NY, USA) and 0.22-µm (Minisart, Sartorius, Göttingen, Germany) syringe filters. Plaques were produced from the filtered sample using the DAL method. Three rounds of plaque purification were conducted for each plaque with a protocol slightly modified from that of Azeredo et al. [34]. Briefly, the plaques were isolated by picking them with a pipette tip and placing them in a saline magnesium (SM) buffer and then centrifuging them at 13,000× *g* for 5 min. The supernatant was analyzed using the DAL method for the production of the next round of purification. Finally, plaques were eluted from the plates in the SM buffer with gentle agitation (90 rpm) for 5 h at 4 °C, and were then centrifuged at 9000× *g* for 10 min at 4 °C and filtered through a 0.22-µm (Minisart, Sartorius, Göttingen, Germany) syringe filter. The filtered JG003 isolate (DSM 19870) phage stock was produced using the DAL method and elution as described above. The phage stocks were stored at 4 °C until use.

### 2.4. Transmission Electron Microscopy (TEM)

Transmission electron microscopy (TEM) was used for the determination of the phage morphology. A filtered high-titer (about 10^11^ PFU/mL) phage stock solution was deposited on a Formvar- and carbon-coated grid (Agar Scientific, Essex, UK) and incubated for 30 s. The excess liquid was removed with a filter paper before the grid was negative stained with phosphor tungstic acid (5%) for 30 s. Phages were examined using a JEM-2100F TEM (JEOL, Tokyo, Japan) in SIB Labs, University of Eastern Finland, Finland.

### 2.5. Phage Host Range

The host range was determined for the three phages (V523, V524 and JG003) using a spot test on a double agar overlay with undiluted phage stock and up to 10^8^ serial diluted phage suspensions (with titers about 10^3^ to 10^11^ PFU/mL). In total, 36 *P. aeruginosa* strains, which included ten strains isolated from clinical samples, 23 strains from environmental samples (three strains from wastewater samples, eight from swimming pool samples and twelve from drinking water tanks) and three reference strains (DSM 19880, DSM 50071 and ATCC 27853) were tested. In addition, non-*P. aeruginosa* bacteria were tested using reference strains of *Pseudomonas fluorescens* (ATCC 49642), *Enterobacter aerogenes* (DSM 30053), *Enterobacter cloacae* (DSM 30054) and three *Escherichia coli* strains (DSM 1103, DSM 30083 and DSM 1576). Aliquots of 10 μL of phage stock or dilution were spotted with a pipette onto the surface of an agar overlay. The plates were incubated for 21 ± 3 h at 36 °C ± 2 °C. The susceptibility of each host to the different phages was evaluated for the appearance of lytic clearing zones, which were classified as clear, turbid or not present.

### 2.6. One-Step Growth Curve

One-step growth curve analyses were performed as previously described [35] to determine the latent periods and burst sizes of the phages. Here, a 20-mL log-phase PAO1 bacterial culture (optical density at 520 nm of 0.24 = 10^8^ CFU/mL) was combined with a phage suspension (10^5^ PFU/mL) to obtain a multiplicity of infection (MOI) of 0.001. The phages were allowed to adsorb for 10 min at 36 °C ± 2 °C, after which the mixture was diluted to 10^−3^. The diluted mixture was incubated at 36 °C ± 2 °C with shaking at 100 rpm. Triplicate 1 mL samples were taken over one hour at 5 min intervals and titrated using the DAL method. The burst size was calculated as the difference between the final and the initial phage titer divided by the initial phage titer.

### 2.7. Phage Genome Sequencing and Assembly

The phage genome was extracted from a 200 μL volume using the High Pure Viral Nucleic Acid Kit according to the manufacturer’s instructions (Roche Molecular Biochemicals Ltd., Mannheim, Germany). The extracted nucleic acids were stored at ≤−75 °C.

The purified DNA was sequenced at SciLife/NGI (Solna, Sweden). A Nextera XT Kit (Illumina Inc., San Diego, CA, USA) was used for the library preparation. Paired-end (2 × 125 bp) sequencing was performed on a HiSeq 2500 instrument (Illumina Inc., San Diego, CA, USA). Raw reads were trimmed to remove adapters and poor quality reads (Trimmomatic version 0.30, settings: -PE –threads 2 -phred33 ILLUMINACLIP:nextera_linkers.txt:2:30:10 LEADING:3 TRAILING:3 SLIDINGWINDOW:4:15 MINLEN:30) [36] and quality was evaluated with FastQC [37]. Trimmed reads were then assembled into contigs using Spades (version 3.6.0, settings: --careful –t 8 --pe1-1 --pe1-2 -o) with a k-mer length of 99 [38]. A first comparison of the genomes to known, previously isolated viruses was conducted with an online basic local alignment search tool, nucleotide, (blastn) against an NCBI nucleotide collection (nr) using the default settings. Given the high similarity of the genomes with phages within the genus *Pbunavirus*, the type-species within this genus, as well as the two phages that V523 and V524 shared the highest similarity with, were downloaded from NCBI for further comparisons (Supplementary Table S1).

An investigation of the taxonomic affiliation of the viruses was conducted using the VIRIDIC [39] and VICTOR [40] online tools with default settings, in which V523 and V524 were compared to the downloaded *Pbunavirus* phages.

Open reading frames (ORFs) were called using GenMark.hmm with Heuristic models [41]. The ORFs were validated through comparison with their closest relatives available in NCBI. Predicted proteins were annotated for function through comparison with the NCBI viral database (ViralDB) created from all viral genomes in the NCBI RefSeq database (July 2019) using BLASTp (v 2.7.1, *e*-value cut-off 0.001). In addition, hmm searches were conducted against the PFAM database (Nov 2020) using the online search tool. The functional annotation, as well as the similarity of V523 and V524 to a closely related *Pbunavirus* phage, were displayed using Easyfig [42].

### 2.8. Phage Biocontrol of P. aeruginosa in Water

Phage biocontrol tests were carried out in 500-mL Erlenmeyer flasks coated with aluminum foil to obtain dark conditions. The test water was the drinking water from the municipality of Siilinjärvi, Finland, which originates from a groundwater source and is disinfected with UV light, using no chlorine prior to distribution. The drinking water was autoclaved prior to tests. The general physical-chemical properties of the drinking water used in this study are presented in Table 1. Phage biocontrol tests were carried out with the *P. aeruginosa* PAO1 strain and the environmental *P. aeruginosa* strain 17V1507. A log-phase bacterial culture was washed twice prior to inoculation, first with a phosphate buffer and then with test water, pelleting the cells by means of centrifugation at 8000× *g* for 2 min. Triplicate 200-mL water samples were inoculated with a 200-µL log-phase *P. aeruginosa* culture (10^5^ CFU/mL) and were kept in agitation (100–125 rpm) at 36 °C ± 2 °C. The phages were spiked in samples after a 24 h adaptation of the host to the test water with the aim of achieving about 10^8^ PFU/mL (MOI of 1000). Triplicate 5 mL samples were taken after 0, 6, 24 (before spike), 24 (after spike), 25, 27, 48, 54 and 72 h from the beginning of the experimental work. Samples were kept in constant agitation (100–125 rpm) and shaken vigorously by hand for 15 s before sampling. Negative controls containing the host without the phage spike were performed for both *P. aeruginosa* strains. Phage controls without the host were also performed for each phage. The samples were immediately subjected to CFU determination of the host bacteria and PFU of phages.

### 2.9. Phage Resistance

The emergence of phage resistance was assessed in the biocontrol study conducted in water. Variation in colony morphologies and increased growth of hosts as the experiment progressed were considered to be due to resistance. To assess the persistence of phage resistance, three consecutive passages on TSA plates were performed for the PAO1 host treated with phage V524. A typical spherical colony was picked from the plate and cultivated on TSA agar plates three times. The resistance of this purified colony was tested against three phages (V523, V524 and JG003) using a spot test on a double agar overlay with undiluted phage stock.

### 2.10. Data Analysis

The statistical analyses were conducted using SPSS, version 25 software for Windows (SPSS Inc., Chicago, IL, USA). Log_10_ reductions were calculated as the average of three replicates. Differences between the average log_10_ reductions of hosts achieved with the individual phages and cocktail were tested using the related-samples Wilcoxon signed-rank test. The method detection limit values were used for the calculations when a below limit of detection result was obtained. Differences were considered significant if the *p*-value was <0.05.

## 3. Results

### 3.1. Phage Isolation and Physical Characterization

**Figure 1:**
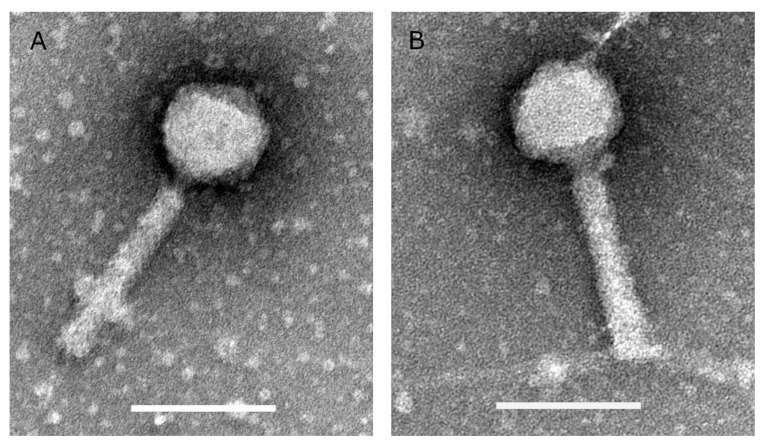
Transmission electron microscopic images of phages (**A**) V523 and (**B**) V524. The scale bars are 100 nm.

Two *P. aeruginosa* phages (V523 and V524) were isolated from wastewater and produced in high titers (1 × 10^11^–8 × 10^11^ PFU/mL). Both phages have isometric heads with a diameter of about 75 nm and contractile tails of about 140 nm (Figure 1).

### 3.2. Phage Host Range

All three phages showed a broad host range against tested *P. aeruginosa* strains (Table 2). Phage JG003 exhibited the broadest activity, lysing 97% of the strains, compared to 67% for phage V523 and 83% for phage V524. The proportions of complete clear lysis zones in spot tests were as follows: 33% for JG003, 14% for V523 and 42% for V524. To verify a positive result, spot tests in dilution series were performed and plaques were obtained in all cases. No cross-reactivity to non-*P. aeruginosa* strains was detected. Spot tests confirmed that all three phages were lytic against PAO1, and for the environmental strain 17V1507 phage V524 and JG003 were lytic.

### 3.3. One-Step Growth Curve

**Figure 2:**
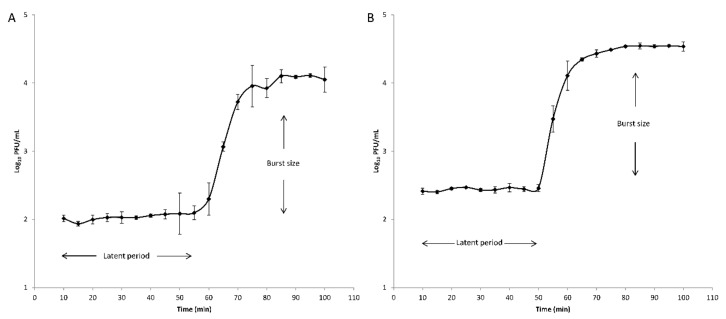
One-step growth curves of the phage (**A**) V523 and (**B**) V524. The error bars represent the standard deviations for technical triplicates. The latent periods and the burst sizes are indicated.

Latent periods and burst sizes were determined for phages V523 and V524 through one-step growth curve analyses using PAO1 as a host (Figure 2). The latent periods were 55 min and 50 min and the burst sizes were 124 PFU/infected cell and 134 PFU/infected cell for phages V523 and V524, respectively.

### 3.4. Phage Genome Characterization

**Figure 3:**
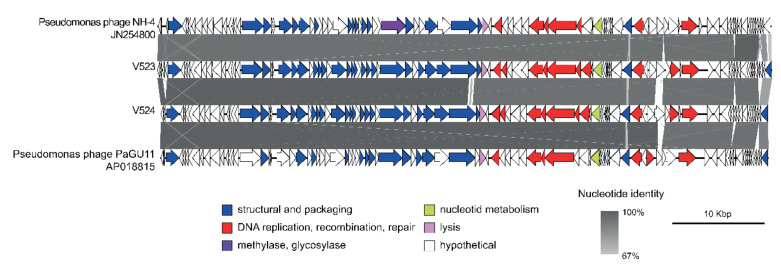
A synteny plot displaying the genome structure of phages V523 and V524, as well as their identity to other closely related *Pbunavirus* phages. The figure was created using Easyfig [42] and the genomes are displayed linearly for visualization. Detailed information regarding the gene annotation can be found in Supplementary Table S1.

**Figure 4:**
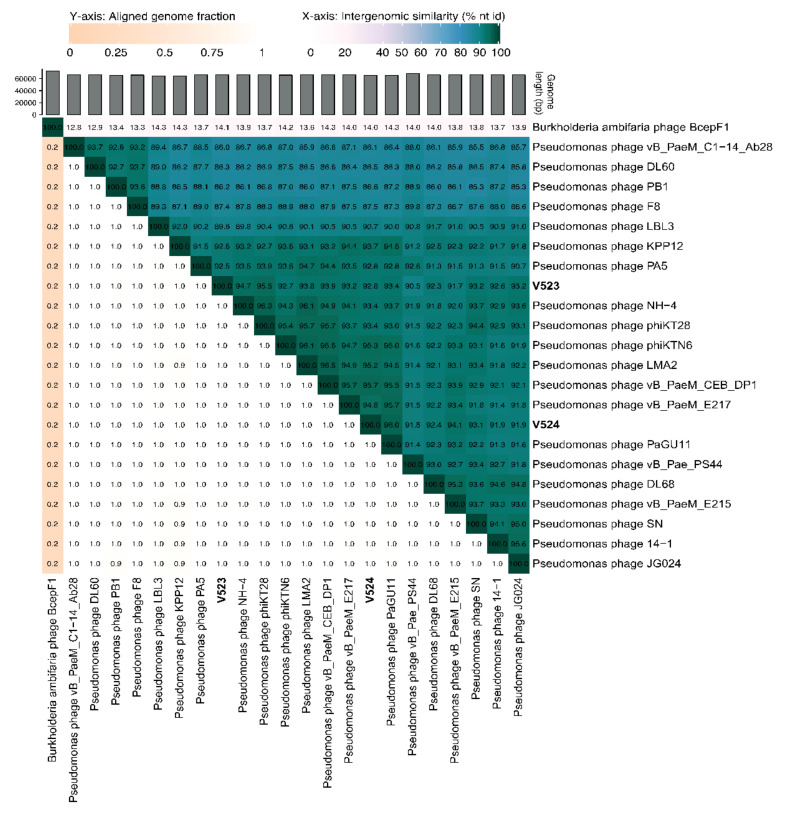
A heat map displaying the similarity between V523 and V524 to reference genomes within the *Pbunavirus* genus and closely related published *Pbunavirus* phage, created using VIRIDIC [39]. Accession numbers for previous phage isolates can be found in Supplementary Table S1.

**Figure 5:**
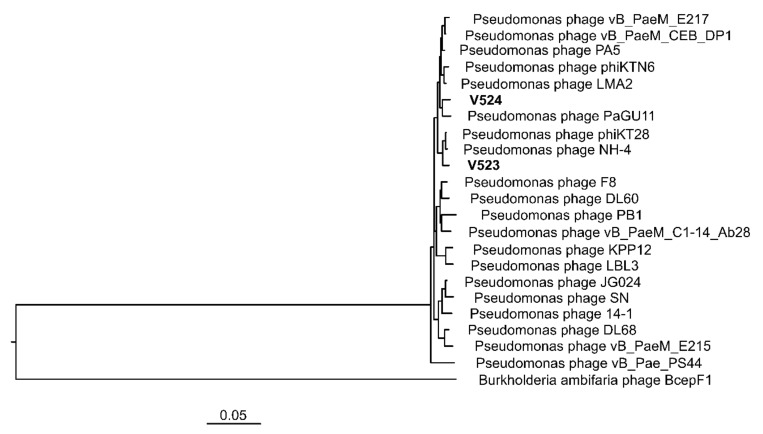
A VICTOR tree produced with FastME based on nucleotide identity calculated with the D0-formula [40], with the newly isolated phages (V523 and V524) and reference genomes within the *Pbunavirus* genus and closely related published *Pbunavirus* phage strains. Accession numbers for previous phage isolates can be found in Supplementary Table S1.

The phages V523 and V524 had genome sizes of 66,301 and 65,447 bp, respectively. They were both assembled into circular genomes, for which the start position of the genome was determined based on a comparison with other closely related phages (Figure 3). The two phages V523 and V524 shared an intergenomic nucleotide identity of 92.8%, identifying them as different species within the same genus (Figure 4). Both V523 and V524 aligned within the *Pbunavirus* genus (Figure 4 and Figure 5). Here, V523 was the most similar to *Pseudomonas* phage phiKT28, sharing an intergenomic nucleotide identity of 95.5%. In turn, V523 and the *Pseudomonas* phage phiKT28 shared 94.7% and 96.3% nucleotide identity, respectively, with Pseudomonas phage NH-4, which is the type phage for the species *Pseudomonas virus NH4*. Phage V524 shared 96.0% nucleotide identity with *Pseudomonas* phage PaGU11. Both V524 and *Pseudomonas* phage PaGU11 shared a high nucleotide identity with *Pseudomonas* phage vB_PaeM_E217 (94.8% and 95.7%, respectively) and *Pseudomonas* phage vB_PaeM_CEB_DP1 (95.7% and 95.5%, respectively).

For V523, 94 ORFs were detected and 90 ORFs were detected for V524. Of these, 38 encoded proteins could be annotated to a putative function within both genomes. These mainly included functions involved in DNA replication and proteins forming the structural virion (Figure 3). Both V523 and V524 contained a predicted thymidylate synthase, which is common among *Pbunaviruses*. No genes suggesting lysogenic replication, antibiotic resistance or toxin production were detected.

### 3.5. Phage Biocontrol of P. aeruginosa in Water

**Figure 6:**
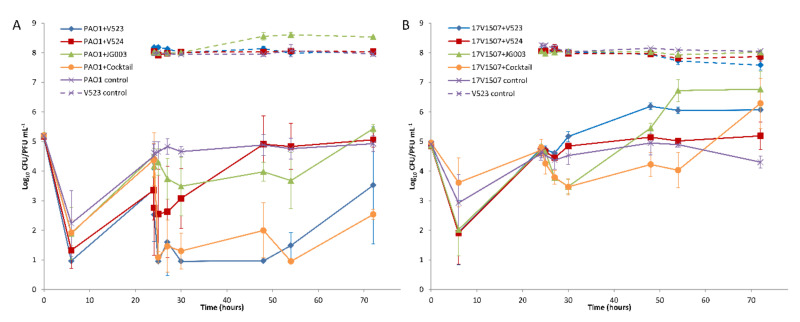
The effect of phages on (**A**) the *P. aeruginosa* PAO1 strain and (**B**) the environmental strain 17V1507. Three separate phages and the phage cocktail were tested. The phages were added after 24 h. Host (solid lines) and phage (dashed lines) numbers were determined from triplicate experiments, and the error bars indicate standard deviations.

**Figure 7:**
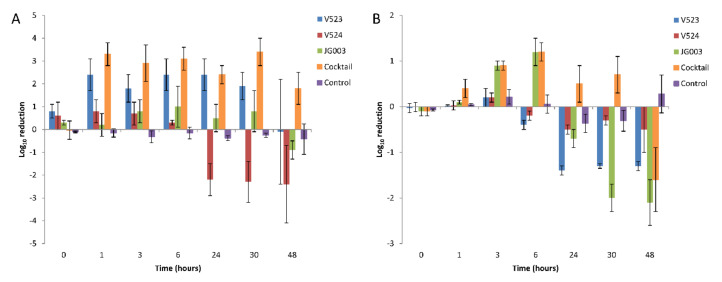
Average log_10_ reductions of (**A**) the *P. aeruginosa* PAO1 strain and (**B**) the environmental strain 17V1507 using bacteriophages in water. Three separate phages and a phage cocktail were tested. Host reductions were determined from triplicate experiments, and the error bars indicate standard deviations.

The hosts were allowed to adapt to the test water for 24 h prior to the addition of the phages. During this time, the numbers of both strains decreased and then recovered to near initial levels (Figure 6), except in tests done with PAO1 treated with phages V523 and V524, in which the levels remained about 1 log lower (Figure 6A). With both host strains, the effect of the phage addition was fast and could be seen within six hours after the application. With the PAO1 strain, phage V523 was the most efficient and produced a statistically significant >2.4 ± 0.7 log_10_ reduction compared to the control (*p* = 0.018, *n* = 7) (Figure 7A). The phage cocktail also produced a statistically significant reduction in the PAO1 strain compared to the control (*p* = 0.018, *n* = 7), with the highest log_10_ reduction of >3.4 ± 0.6 measured 30 h post-infection. The log_10_ reductions achieved with phage V523 and the cocktail did not differ statistically. The reductions observed with phage V524 and JG003 were restored and the phage counts returned to near-control levels within two days (Figure 6A).

With the environmental 17V1507 host, phage JG003 was the most efficient of the individual phages, showing a 1.2 ± 0.3 log_10_ reduction within six hours (Figure 6B and Figure 7B). The phage cocktail achieved an equal reduction within six hours (1.2 ± 0.2). The reductions in the 17V1507 strain observed with phage JG003 and the cocktail were restored, and the numbers ended up being higher compared to the control levels. The replication of the 17V1507 host was slower in the cocktail compared to JG003, as evidenced by the significant statistical difference between the log_10_ reductions of phage JG003 and the cocktail (*p* = 0.043, *n* = 7). The slight reductions observed with phage V523 and V524 were restored and the host counts ended up being higher compared to the control.

The PAO1 strain was more susceptible than the environmental 17V1507 strain to phage V523 (*p* = 0.018, *n* = 7) and the phage cocktail (*p* = 0.018, *n* = 7).

### 3.6. P. aeruginosa Resistance

The hosts treated with phages produced resistance during the experiment. Initially, the colonies produced on TSA plates showed variations in size and morphology (small colonies with irregular shapes) but became more regular in shape as the experiment progressed. The use of the phage cocktail reduced the development of resistance with both hosts compared to the use of individual phages (Figure 6 and Figure 7). The PAO1 resistance to phage V524 was stable after three consecutive passages on TSA plates. Resistance to phage V524 did not protect the PAO1 host against the other two phages (V523 and JG003).

## 4. Discussion

In this study, two *P. aeruginosa* phages were isolated from wastewater. The physical and genome characterization revealed that these phages belonged to the genus *Pbunavirus*, which constitute environmentally very widespread phages within the Myoviridae family [43]. The two isolated phages (V523 and V524) showed high similarity in their morphology, growth characteristics (latent period and burst size) and genome structure. The sequence identity between phages V523 and V524 was 92.8%. Phage V523 shared the highest nucleotide identity (95.5%) with *Pseudomonas* phage phiKT28 [44] and phage V524 had the highest identity with *Pseudomonas* phage PaGU11 (96%) (accession: AP018815). In turn, *Pseudomonas* phage phiKT28 and V524 and *Pseudomonas* phage PaGU11 shared >95% nucleotide identity with one and two phages, respectively, that represent an individual viral species within the *Pbunavirus* genus. A 95% nucleotide identity is set as the cut-off defining the species of the phages [45]. However, the micro-diversity among the *Pseudomonas* phages (Figure 4) makes it difficult to define which species V523 and V524 belong to, as several of the species share >95% nucleotide identity between each other. The genomes of V523 and V524, like the *Pbunavirus* phages we compared them to (Figure 3) [46], do not encode a recognizable integrase, toxin genes or antibiotic resistance genes, supporting their suitably for application in phage therapy experiments.

The studied phages were lytic against a broad range of *P. aeruginosa* hosts. The phage infectivity, tested with clinical and environmental *P. aeruginosa* strains (*n* = 36), revealed that 67% and 83% of the hosts were sensitive to phage V523 and V524, respectively. JG003 had the broadest host range of the three phages, lysing 97% of the strains. JG003 belongs to the *ph*A group (phenotypically) and originates from sewage [47] (no genome data available). For the biocontrol applications, lytic phages with a broad host range are preferred and recommended. Moreover, growth characteristics are important determinants for efficient biocontrol, and in our study, a longer latent period (55 vs. 35 min), but a much higher burst size (124 vs. 65 PFU/infected cell) was observed with phage V523 in comparison to its closest relative phage KT28 [44]. Overall, the large burst size and relatively short latent period are desirable characteristics for a phage used in biocontrol applications [48].

This study describes the potential of using bacteriophages as biocontrol agents in water. The results showed that phages can control the growth and numbers of planktonic bacteria in water. The interaction was relatively fast and the highest log reductions were observed within 6 h. Differences in efficiency were noted between the phages against the two *P. aeruginosa* hosts in water. Of the three phages, V523 produced a significant >2.4 log_10_ reduction in the numbers of the PAO1 host during the 48-h challenge test. However, the reduction was reversed between 30 h and 48 h after the addition of V523. A similar loss of reduction was not observed with the phage cocktail. Phage JG003 caused a reduction in the numbers of both hosts (PAO1 and 17V1507, 1.0 and 1.2 log_10_ reductions, respectively) during the first 6 h after the spike, but the counts were restored during the following 48 h. Phage V524 had a poor effect in the biocontrol study. A cocktail of three phages showed a slightly higher decrease in the level of the hosts compared to the use of individual phages. The highest observed log_10_ reduction of >3.4 was achieved with a cocktail of three phages in the PAO1 host. The greater reduction of the PAO1 observed with the cocktail (>3.4 log) than with the phage V523 (>2.4) was probably due to the higher initial number of hosts before the spike in the test carried out with the cocktail. However, no clear synergistic effect was observed with the use of the cocktail, i.e., the cocktail did not produce higher reductions compared to the sum of the reductions of the three individual phages. Synergy has been described as one important reason for using cocktails in phage therapy [49].

It has been assumed that planktonic cells are easier targets for phage control than cells protected by biofilms [50,51]. A previous study showed that phages can eliminate up to 90% of *P. aeruginosa* strains (including PAO1) in a liquid medium [52]. More studies have been carried out with *P. aeruginosa* biofilms (reviewed in Chegini et al. [53]). Pires et al. [54] demonstrated that the administration of bacteriophages to bacterial biofilm cultures achieved a 2–3 log_10_ reduction in the *P. aeruginosa* cell number. Similarly, Magin et al. [52] showed a maximum reduction of 1.7 log_10_ in a biofilm, using viability-qPCR. These reductions are consistent with our results.

The reference strain PAO1 was more susceptible to the phages than the environmental strain 17V1507. This is consistent with the spot test results, in which PAO1 produced clear lysis zones for all three phages, and as with the 17V1507 strain, turbid plaques were observed with phage V524 and JG003. PAO1 also showed higher variation than 17V1507 between the replicates during the 72-h tests in water. These observations may be partly due to the different origin of the PAO1 strain (an infected wound) compared to the environmental strain 17V1507, isolated from swimming pool water. With strain 17V1507, better results could have been achieved with isolating phages with higher activity against this environmental isolate. Overall, the results clearly indicate that the careful selection of individual phages against a target host and the design of the phage cocktail are important. Moreover, the use of a same phage cocktail for different host strains is not recommended prior to further testing.

The results clearly show that bacteria can develop phage resistance in water. In this study, resistance was evident, since even though the colony counts of the bacterial hosts decreased (even to below detection-limit values) after phage addition, growth of the hosts was observed towards the end of the test period. This growth in spiked waters may be favored by the nutrients that came with the phage spike, since in the controls this growth was not observed. The emergence of phage resistance has been detected previously with *P. aeruginosa* [44,54,55]. Our study indicated that the resistance was stable after three consecutive passages on TSA plates. It was noteworthy that PAO1 resistance to phage V524 did not protect the host against other two phages (V523 and JG003). However, phage resistance usually comes at the cost of cell fitness [56,57]. In the present study, this was observed on the agar plates, which initially produced atypical colony morphologies (smaller colonies and/or a star-like shape), but later normal colonies (round) increased on the plates. In clinical settings, it is assumed that the host does not need to be killed totally since the immune system will take care of weakened pathogens. This is a prerequisite for a successful phage therapy because the total destruction of the host is rarely possible. Positive results have been documented, e.g., using phages in the treatment of antibiotic-resistant *P. aeruginosa* in chronic otitis [58]. There are also reports showing synergistic effects using phages and antibiotics in medical applications [59,60]. Similarly, in water environments, the phage-treated weakened pathogens are thought to be more susceptible to disinfectants [61]. This could allow the use of lower levels of chemicals and could enable the efficient disinfection of microbes which are tolerant of chemicals. The use of the phage cocktail clearly reduced the development of resistance. Therefore, the use of a cocktail is highly recommended in future applications in water.

## 5. Conclusions

The encouraging results obtained with the *P. aeruginosa* biocontrol in water emphasize the potential of using phages to control harmful bacteria. New control options are needed since antibiotic-resistant bacteria, such as multidrug-resistant *P. aeruginosa*, may spread in water environments and become a potential health threat if they are present in drinking water or recreational water such as in swimming pools and spas. Biocontrol in water may be sufficient to reduce the number of pathogenic bacteria below acceptable levels, at which there is no expected risk to health, and to inhibit their growth. However, more knowledge is needed in several respects related to phage use as a biocontrol agent in water, including the safety, efficiency, resistance, use and design of phage cocktails, the use of phage and disinfectants together and the role of matrices (water vs. biofilms). The main conclusions of this study are as follows:

1. Lytic *P. aeruginosa* phages with a broad host range were isolated and characterized for biocontrol purposes in water.
2. Tests conducted in water showed differences between the efficiency of phages and the sensitivity of hosts.
3. A three-phage cocktail showed more consistent and efficient reduction of hosts compared to the use of a single phage.
4. Phage resistance appeared rapidly and its control requires further studies.
5. Careful design of the phage cocktail is important in order to achieve maximum reductions and to curtail possible phage resistance.

## Figures and Tables

**Table 1:** Averages and standard deviations of physical-chemical properties of the test water (*n* = 4).

Parameter	Drinking Water
pH	7.3 ± 0.3
Conductivity, μS cm^−1^	446 ± 8
AOC ^1^, μg AOC-C L^−1^	113 ± 70
AOC, μg acetate-C L^−1^	74 ± 47
MAP ^2^, μg MAP-P L^−1^	1.5 ± 0.6

^1^ AOC, assimilable organic carbon; ^2^ MAP, microbially available phosphorus.

**Table 2:** Host range of individual phages V523, V524 and JG003.

Host Strain	Source	V523	V524	JG003
PAO1 (DSM 19880)	Clinical	++	++	++
17V1507	Swimming pool	−	+	+
17V1508	Swimming pool	−	+	+
17V1509	Swimming pool	−	++	++
17V1510	Swimming pool	−	+	++
17V1511	Swimming pool	−	+	+
17V1512	Swimming pool	−	+	++
18V0528/1b	Swimming pool	+	+	+
19V2041	Swimming pool	+	+	++
17V427/2a	Wastewater	+	++	+
17V427/7a	Wastewater	+	++	+
17V427/9a	Wastewater	+	++	+
19V2030	Water tank	+	++	+
19V2031	Water tank	+	++	+
19V2032	Water tank	+	++	+
19V2033	Water tank	+	+	+
19V2034	Water tank	+	+	+
19V2035	Water tank	−	++	++
19V2036	Water tank	+	++	+
19V2037	Water tank	+	++	+
19V2038	Water tank	+	++	+
19V2039	Water tank	++	+	+
19V2040	Water tank	+	+	++
19V2042	Water tank	+	++	+
19V1974	Clinical	−	++	++
19V1976	Clinical	+	−	+
19V1977	Clinical	+	−	++
19V1978	Clinical	−	−	++
19V1979	Clinical	++	+	−
19V1980	Clinical	++	−	++
19V1981	Clinical	++	−	+
19V1982	Clinical	+	−	++
19V1983	Clinical	+	++	+
19V1984	Clinical	−	+	+
ATCC 27853	Clinical	−	+	+
DSM 50071	Not known	−	+	+
# of sensitive strains		24/36 (67%)	30/36 (83%)	35/36 (97%)
# of sensitive strains (clear)		5/36 (14%)	15/36 (42%)	12/36 (33%)

++, clear plaque (complete lysis); +, turbid plaque (partial lysis); −, no plaque (no lysis); #, number.

## Data Availability

The data presented in this study are included in the article and the Supplementary Materials. The viral genomes were deposited in the NCBI GenBank with the accession numbers MW595220 and MW595221.

## Supplementary Materials

The following are available online at https://www.mdpi.com/article/10.3390/v13050928/s1, Table S1: Information regarding the accession number and species affiliation for the *Pbunavirus* phages used for genome comparison in Figure 3, Figure 4 and Figure 5. Table S2: Information about the gene arrangement and functional annotation of the two phages, V523 and V524, as well as best match to viralDB and PFAM.
