# Supplementary material for: Phage Biocontrol of Pseudomonas aeruginosa in Water
Source: Viruses. 2021 May 17;13(5):928. doi: 10.3390/v13050928 (PMC8156286; doi:10.3390/v13050928)
Supplement: Supplementary file 1 [file viruses-13-00928-s001.zip › Table S1.pdf]

Table S1. Information regarding the accession number and species affiliation for the *Pbunavirus* phages used for genome comparison in Figures 3-5.

Phage isolate	Accession number	Defined species
Pseudomonas phage 14-1	NC_011703	*Pseudomonas virus 141*
Pseudomonas phage vB_PaeM_C1-14_Ab28	NC_026600	*Pseudomonas virus Ab28*
Pseudomonas phage vB_PaeM_CEB_DP1	NC_041870	*Pseudomonas virus CEBDP1*
Pseudomonas phage DL60	NC_028745	*Pseudomonas virus DL60*
Pseudomonas phage DL68	NC_028971	*Pseudomonas virus DL68*
Pseudomonas phage vB_PaeM_E215	NC_042080	*Pseudomonas virus E215*
Pseudomonas phage vB_PaeM_E217	NC_042079	*Pseudomonas virus E217*
Pseudomonas phage F8	NC_007810	*Pseudomonas virus F8*
Pseudomonas phage JG024	NC_017674	*Pseudomonas virus JG024*
Pseudomonas phage KPP12	NC_019935	*Pseudomonas virus KPP12*
Pseudomonas phage phiKTN6	NC_041865	*Pseudomonas virus KTN6*
Pseudomonas phage LBL3	NC_011165	*Pseudomonas virus LBL3*
Pseudomonas phage LMA2	NC_011166	*Pseudomonas virus LMA2*
Pseudomonas phage NH-4	JN254800	*Pseudomonas virus NH4*
Pseudomonas phage PA5	NC_041902	*Pseudomonas virus PA5*
Pseudomonas phage PaGU11	AP018815	no defined species
Pseudomonas phage PB1	EU716414	*Pseudomonas virus PB1*
Pseudomonas phage phiKT28	KP340287	no defined species
Pseudomonas phage vB_Pae_PS44	NC_028939	*Pseudomonas virus PS44*
Pseudomonas phage SN	FM887021	*Pseudomonas virus SN*
Burkholderia ambifaria phage BcepF1	NC_009015	*Burkholderia virus BcepF1*
